# Comparison between the accuracy of conventional and portable computed tomography-based navigation systems in total hip arthroplasty

**DOI:** 10.1007/s00402-026-06321-4

**Published:** 2026-04-26

**Authors:** Hiroshi Asai, Yusuke Osawa, Yasuhiko Takegami, Hiroto Funahashi, Shiro Imagama

**Affiliations:** Nagoya University, Nagoya, Japan

**Keywords:** Acetabular cup orientation, Hip osteoarthritis, Computer-assisted surgery, Portable computed tomography-based navigation, Computed tomography-based navigation, Total hip arthroplasty

## Abstract

**Background:**

Although conventional computed tomography (CT)-based navigation provides excellent placement accuracy and clinical outcomes, whether recently introduced portable systems can achieve comparable results remains unclear. This study aimed to evaluate the placement accuracy and surgical outcomes of portable CT-based navigation systems.

**Methods:**

This study assessed 56 hips of patients that underwent total hip arthroplasty (THA) using portable CT-based navigation. Using propensity score matching based on age, sex, and body mass index, we identified 51 hips treated with portable CT-based navigation (portable CTN group) and 51 hips with conventional CT-based navigation (CTN group). The evaluation parameters included cup orientation accuracy, cup positioning, operative time, blood loss, preoperative and postoperative Japanese Orthopaedic Association scores, and complications.

**Results:**

Regarding accuracy error, the portable CTN (radiographic inclination [RI]: 2.8 ± 2.8°, radiographic anteversion [RA]: 3.8 ± 3.2°) and CTN groups (RI: 2.7 ± 1.9°, RA: 3.0 ± 2.1°) did not significantly differ. For navigation error, the portable CTN group (RI: 3.2 ± 3.2°, RA: 3.5 ± 3.1°) had significantly inferior results to the CTN group (RI: 2.2 ± 1.7°, RA: 2.3 ± 1.7°) regarding anteversion. The portable CTN group demonstrated a significantly lower accuracy, as the proportion of hips with a navigation error of > 5° was 31.4% (16 hips), compared to 11.8% (6 hips) in the CTN group. For cup position, the horizontal position error in the portable CTN group was 3.1 ± 1.8 mm, significantly less accurate than the 2.3 ± 1.9 mm in the CTN group. No significant differences were observed in the clinical outcomes within the follow-up period of one year.

**Conclusion:**

Portable CT-based navigation was inferior to conventional CT-based navigation in terms of placement accuracy, including cup orientation and positioning. While clinical outcomes did not significantly differ at the one-year follow-up, this short-term follow-up limits conclusions on long-term clinical equivalence. Future advancements in portable CT-based navigation systems are required to improve their accuracy.

## Introduction

 Total hip arthroplasty (THA) is a common surgery for end-stage hip disease, with established high clinical efficacy and patient satisfaction [1, 2]. Precise implant placement is crucial for preventing dislocation, impingement, and polyethylene wear [3, 4]. Dislocation remains one of the leading causes of revision surgery [5]. Therefore, various computer-assisted surgical systems have been developed to improve the accuracy of implant placement. Particularly, computed tomography (CT)-based navigation systems have been proven to provide excellent accuracy regarding alignment angles and positioning, even in complicated cases [6–8].

Conventional CT-based navigation systems consist of an integrated unit, including a main console, display, and infrared camera, complicating their transportation. Furthermore, high implementation costs have limited their availability in low resource settings. To address these issues, a portable CT-based navigation system featuring a handheld device with a portable infrared camera and display has recently been developed. However, whether portable CT-based navigation can achieve an accuracy comparable to that of conventional systems remains unclear, given the potential limitations of the infrared camera’s field of view, precision, and computer processing speed. Additionally, while portable systems utilize surface matching similar to conventional ones, the risk that decreased registration accuracy could necessitate re-registration may prolong the operative time.

The clinical questions of this study were: (1) Does the portable CT-based navigation system provide accuracy comparable to that of the system? (2) Is the reproducibility of the preoperative plan for implant positioning equivalent when using a portable CT-based navigation system? (3) Do the operative time or clinical outcomes significantly differ between portable and conventional CT-based navigation?

## Materials and methods

### Patients

**Fig. 1:**
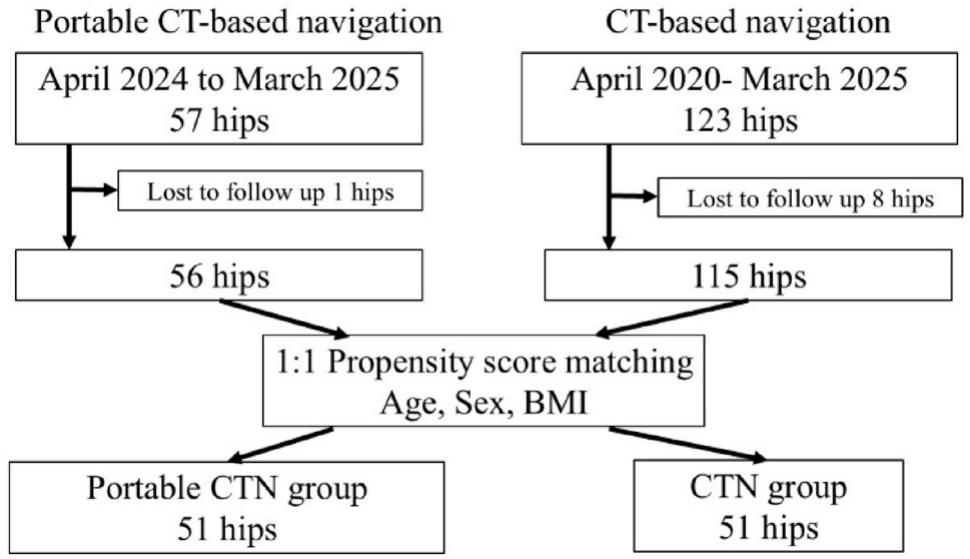
Study design. BMI, body mass index

This retrospective study was approved by the Institutional Review Board. All patients provided informed consent prior to inclusion in the study. The study assessed 57 hips from patients that underwent THA for Crowe Type I hip osteoarthritis using a portable CT-based navigation system (portable CTN group) between April 2024 and March 2025, of which 56 were followed up for more than 1 year. For comparison, 123 hips from patients that underwent THA for Crowe Type I hip osteoarthritis using a conventional CT-based navigation system (CTN group) between April 2020 and March 2025 were identified, with 115 followed up for more than 1 year used as the control group. During the study period, patients were allocated to one of four available navigation systems, comprising two portable systems (CT-based navigation, the latter introduced in April 2024 and AR navigation) and one conventional system (CT-based navigation), and one robotic-assisted system, in a quasi-random manner. Propensity score matching was performed to address potential confounding factors. A 1:1 matched cohort was created based on age, sex, and body mass index (BMI), with the caliper width set at 0.2. Consequently, the final analysis included 51 hips in each of the portable CTN and CTN groups (Fig. 1).

The portable CTN group consisted of 47 patients (51 hips), including seven men (seven hips) and 40 women (44 hips), whereas the CTN group comprised 49 patients (51 hips), including eight men (eight hips) and 41 women (43 hips). The mean age was 66.7 years (range: 49–84 years) in the portable CTN group and 67.5 years (range: 49–86 years) in the CTN group. The mean follow-up periods were 15.6 months (range, 12–24) and 46.6 months (range, 12–72 months), respectively (Table 1). The Trident (Stryker, Mahwah, Michigan, USA) implants were used in all patients in the portable CTN group, whereas SQURAM (KYOCERA, Kyoto, Japan) implants were used in all patients in the CTN group. The mean cup sizes were 50.0 ± 2.4 mm and 50.2 ± 2.4 mm, and the mean number of screws used was 1.8 ± 0.6 and 1.9 ± 0.7, respectively. In the portable CTN group, Secur-Fit Advanced (Stryker) stems were used in 45 cases and Exeter in 6, while INITIA (KYOCERA) stems were used in all the CTN group cases (Table 1).

**Table 1:** Characteristics of the patients

	Portable CTN group	CTN group	*P*-value
Number of hips	51	51	
Number of patients	47	49	
Age (years)	66.7 ± 7.9	67.5 ± 8.9	0.64
Sex (men/women)	7/44	8/43	> 0.99
BMI (kg/m^2^)	24.6 ± 4.6	24.1 ± 4.6	0.53
Crowe classification			
Ⅰ	51	51	> 0.99
Cup	Trident 51	SQRUM 51	
Cup size (mm)	50.0 ± 2.4	50.2 ± 2.4	> 0.99
Number of screws	1.8 ± 0.6	1.9 ± 0.7	0.44
Stem	Secur-fit advanced 45	INITIA 51	
	Exeter 6		
Follow up period (months)	15.6 ± 4.2	46.6 ± 8.0	< 0.001

CTN, computed tomography-based navigation; BMI, body mass index

### Surgical procedure

Preoperative planning was performed using a three-dimensional (3D) templating software (ZedHip, Lexi, Tokyo, Japan). The cup was positioned at an anatomical height, and cementless cups were selected when a cup center-edge angle of ≥ 0° was achievable [9]. Cementless cups were used in all cases. All THA procedures were performed using the posterior approach in the lateral decubitus position. Target al.ignment was set based on the functional pelvic plane (FPP) in the supine position, based on both the anterior superior iliac spines and gravity axis [10, 11]. The targets were set to achieve a radiographic inclination (RI) of 40° and radiographic anteversion (RA) of 20°, according to Murray’s definition [12]. All surgical procedures were performed by a total of seven orthopedic surgeons at our institution. Intraoperatively, the surgeon determined the use of screws for cup fixation. Postoperatively, full weight bearing was permitted, and gait training was initiated. One week postoperatively, CT was performed to evaluate the accuracy of the implant’s orientation and position.

### Navigation systems

#### Portable CT-based navigation

**Fig. 2:**
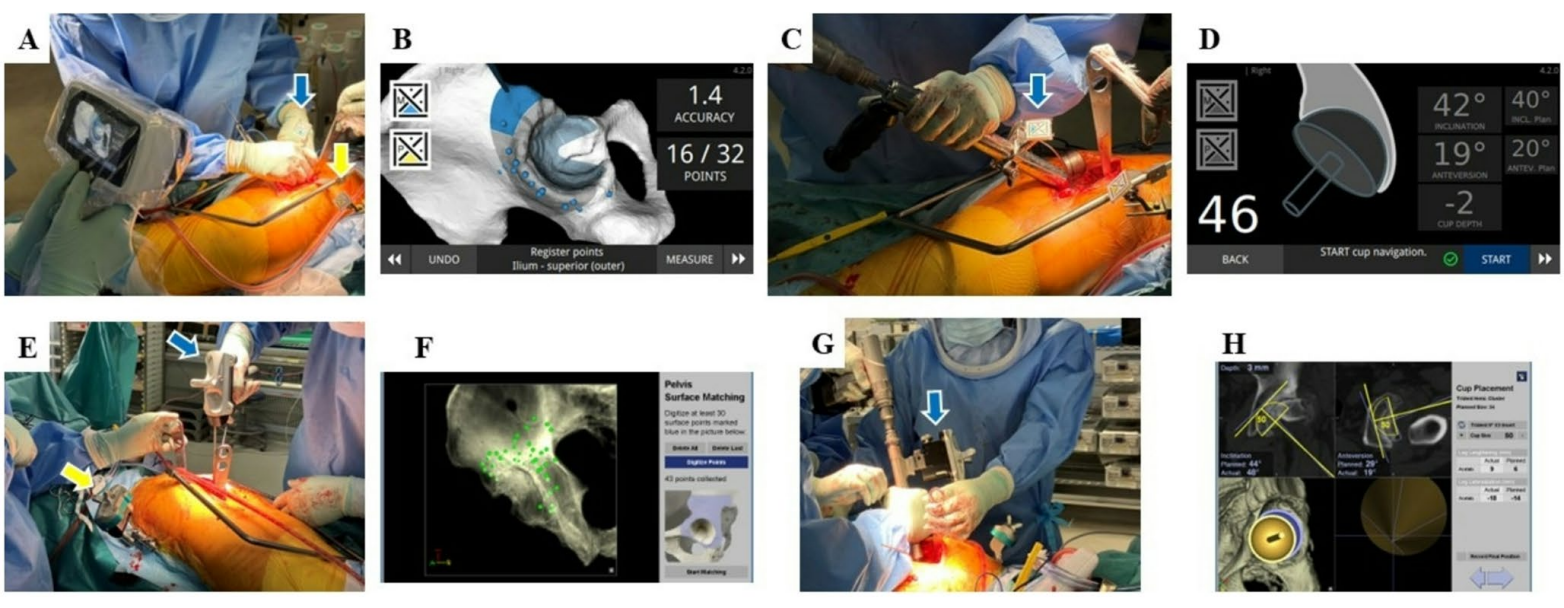
Portable CT-based navigation system and CT-based navigation system. **A** Portable CT-based navigation system. Intraoperative image during registration. The pelvic surface is palpated with a pointer with a blue tag (Indicated by the blue arrow). A yellow tag is attached to the pelvis (Indicated by the yellow arrow). **B** Navigation screen during surface matching. **C** The blue tag is attached to the reamer (Indicated by the blue arrow). **D** Navigation screen during reaming and cup placement. The angle and position of the cup was checked. **E** CT-based navigation system. Intraoperative image during registration. The pelvic surface is palpated with a pointer (Indicated by the blue arrow). The tracker is attached to the pelvis (Indicated by the yellow arrow). **F** Navigation screen during surface matching. **G** Navigation attached to the reamer (Indicated by the blue arrow). **H** Navigation screen during reaming and cup placement. The angle and position of the cup was checked

Naviswiss Hip CT (Naviswiss AG, Brugg, Switzerland) was used as a portable CT-based navigation system. The pelvis was segmented, and the coordinates were defined based on anatomical reference points. Prior to skin incision, the pelvic tracker was secured to the pelvis using pins. Intraoperative pelvic surface matching was performed using the portable infrared stereo camera, involving the registration of 32 points on the bone surface. Following surface matching, registration accuracy was verified through measuring the residual errors at representative points on the anterior wall, posterior wall, and superior aspect of the acetabulum. Intraoperative navigation was used to adjust and guide the final orientation and position of the acetabular cup (Fig. 2).

#### Conventional CT-based navigation

The CT-based hip navigation system Stryker (Mahwah, Michigan, USA) was used. The pelvis was segmented and coordinates were established using anatomical reference points. Similar to the protocol for portable CT-based navigation, a pelvic tracker was attached to the pelvis prior to skin incision. Intraoperatively, pelvic surface matching was performed by registering more than 30 points. The accuracy of the registration was verified using a pointer to confirm representative anatomical landmarks. Finally, the intraoperative navigation system was used to adjust and guide the orientation and positioning of the acetabular cup during implantation [6, 13].

### Postoperative evaluations

Postoperative evaluations were performed using a 3D templating software (ZedHip, Lexi, Tokyo, Japan). All radiographic measurements were performed in a blinded manner. The orientation and position of the acetabular cup were assessed through superimposing a 3D template onto the actual implanted cup [13, 14]. RI and RA were measured relative to the FPP in the supine position. The reported intra-observer correlation coefficients of this method were 0.976 for RI and 0.966 for RA, while the inter-observer correlation coefficients were 0.959 and 0.918, respectively [15]. An accuracy error was defined as the difference between the target angles (RI 40° and RA 20°) and the actual postoperative angles. A navigation error was calculated as the difference between the angles displayed on the navigation screen during surgery and the actual postoperative angles. Both values were calculated as absolute errors. Furthermore, for navigation errors, cases in which either the RI or RA were deviated by ≥ 5° or ≥ 10° were defined as outliers, and their proportions were calculated.

**Fig. 3:**
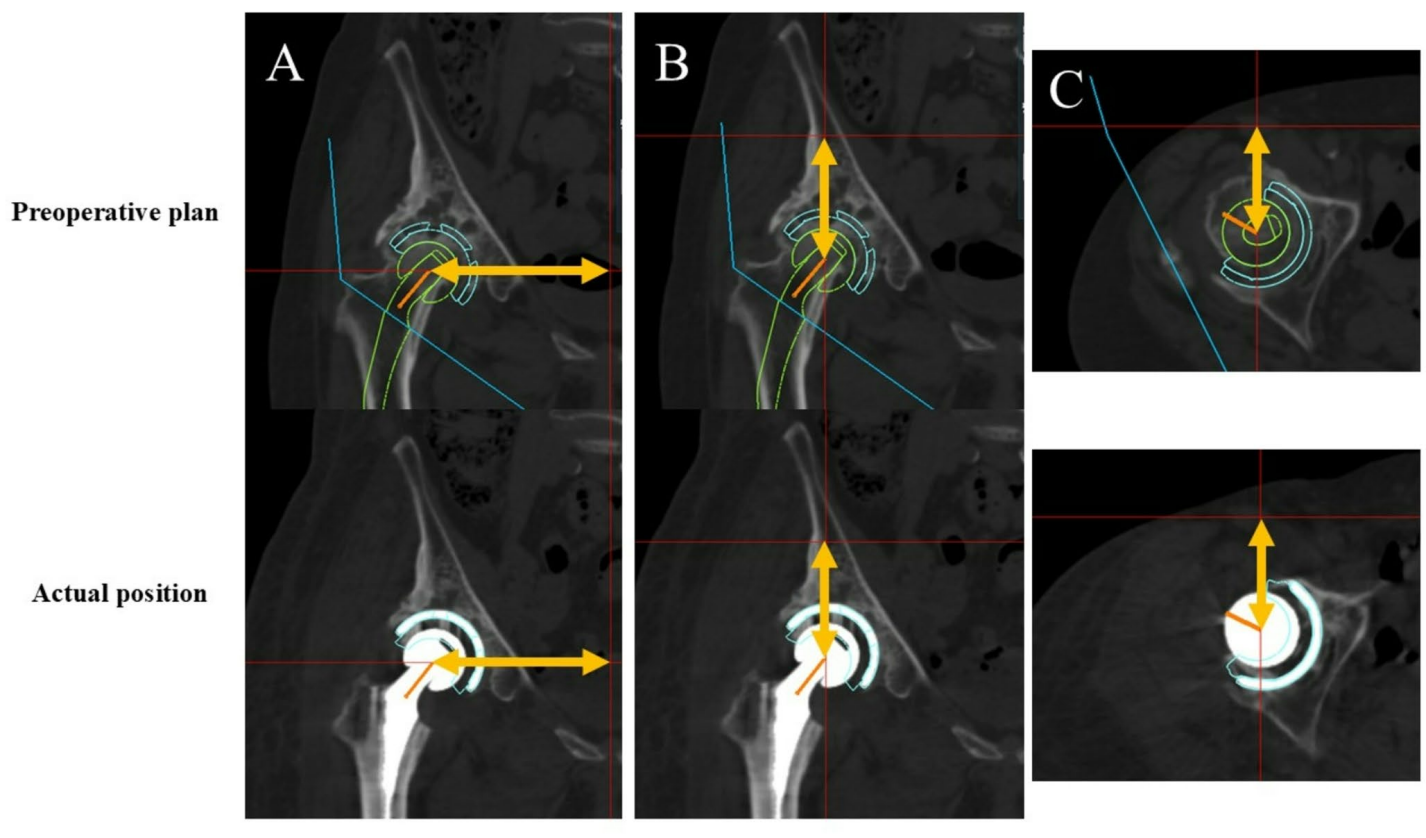
How to measure the cup position in each direction. We measured the cup position of preoperative plan and actual position, and calculated the gaps in each direction. **A** Horizontal direction. **B** Vertical direction. **C** Anteroposterior direction

The acetabular cup position was evaluated using a 3D coordinate system based on the anterior pelvic plane (APP). The APP was defined by the bilateral anterior superior iliac spines (ASIS) and the pubic symphysis [16]. The origin of the coordinate system was set at the midpoint between the bilateral ASIS. The horizontal direction was defined by the line connecting the bilateral ASIS, the anteroposterior direction was defined as the axis perpendicular to the APP, and the vertical direction was defined as the axis perpendicular to both the x and y axes. The distance from the origin to the center of the acetabular component was measured along each of these three axes, both in the preoperative planning and postoperatively. The errors between the preoperative plan and the actual postoperative cup position were calculated and defined as horizontal, vertical, and anteroposterior position errors [6, 14, 16, 17] (Fig. 3).

### Surgical outcomes

The operative time, intraoperative blood loss, and intraoperative and postoperative complications, including dislocation, infection, intraoperative and postoperative fractures, and nerve injury, were recorded. In addition, the Japanese Orthopaedic Association (JOA) scores were assessed preoperatively and 1 year postoperatively.

### Data analyses

Based on the previous report with a standard deviation of 2.8°, a power analysis determined that a sample size of 42 hips per group would be required to detect a navigation error of 2° with an α level of 0.05 and a power of 90% [18]. Continuous and categorical variables were analyzed using the independent-samples *t*-test and Fisher’s exact test, respectively. For variables that deviated from a normal distribution, the Mann-Whitney U test was employed. All statistical analyses were performed using EZR (Saitama Medical Center, Jichi Medical University, Saitama, Japan), and statistical significance was considered at *P* < 0.05.

## Results

**Fig. 4:**
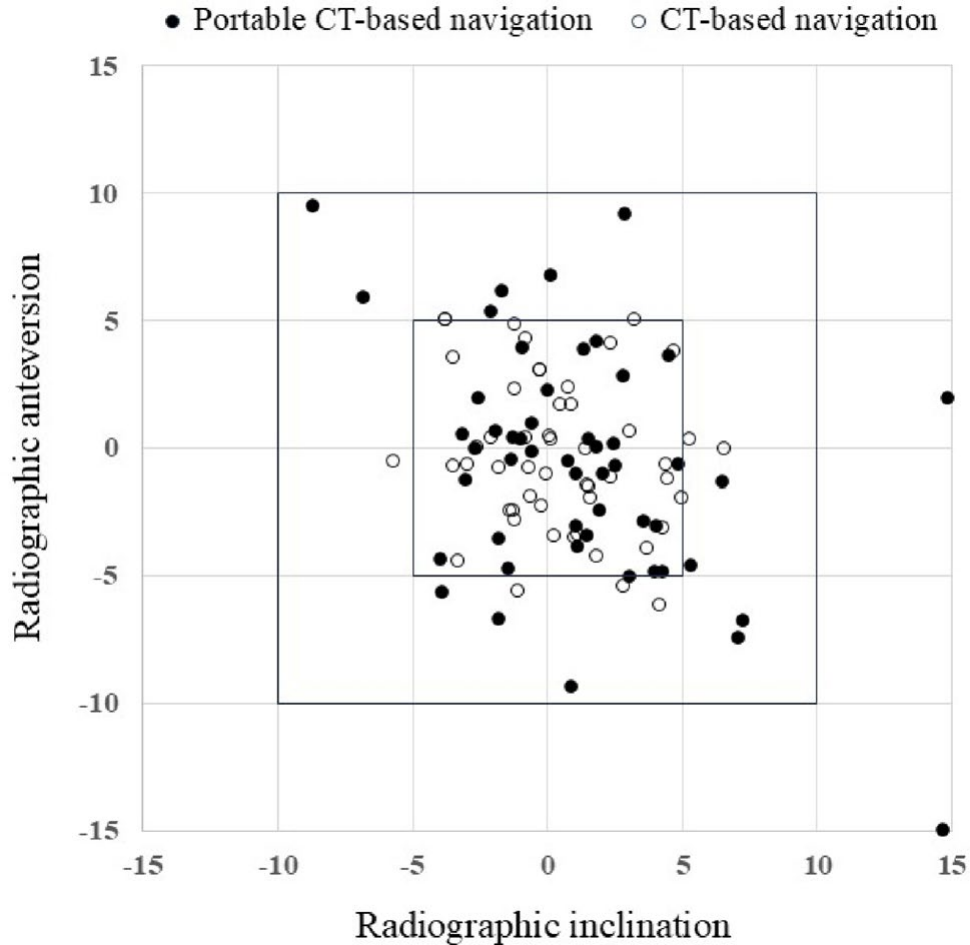
The scatterplots of the navigation errors. Horizontal axis represents the navigation error in radiographic inclination, and vertical axis represents the navigation error in radiographic anteversion

The mean postoperative cup orientation in the portable CTN group was RI 41.2 ± 3.7° and RA 18.7 ± 4.8°, while in the CTN group, it was RI 39.8 ± 3.3° and RA 18.9 ± 4.0°. Regarding accuracy errors, the values were RI 2.8 ± 2.8° and RA 3.8 ± 3.2° in the portable CTN group, and RI 2.7 ± 1.9° and RA 3.0 ± 2.1° in the CTN group. The navigation error in the portable CTN group was RI 3.2 ± 3.2° and RA 3.5 ± 3.1°, whereas in the CTN group, it was RI 2.2 ± 1.7° and RA 2.3 ± 1.7°. The navigation error for the RA was significantly larger in the portable CTN group (*P* = 0.015). Furthermore, a navigation error of ≥ 5° was significantly higher in the portable CTN group, observed in 16 hips (31.4%), compared to 6 hips (11.8%) in the CTN group (Table 2; Fig. 4).

**Table 2:** Radiographic outcome

	Portable CTN group	CTN group	*P*-value
Cup placement angles measured postoperatively			
RI (°)	41.2 ± 3.7	39.8 ± 3.3	0.14
RA (°)	18.7 ± 4.8	19.2 ± 3.6	0.52
Cup placement angles displayed on the navigation screen			
RI (°)	40.0 ± 3.3	39.3 ± 2.6	0.17
RA (°)	19.4 ± 3.6	19.2 ± 3.4	0.79
Absolute value of accuracy error			
RI (°)	2.8 ± 2.8	2.7 ± 1.9	0.38
RA (°)	3.8 ± 3.2	3.0 ± 2.1	0.14
Absolute value of navigation error			
RI (°)	3.2 ± 3.2	2.2 ± 1.7	0.12
RA (°)	3.5 ± 3.1	2.3 ± 1.7	0.015
Outlier > 5°	16 (31.4%)	6 (11.8%)	0.029
Outlier > 10°	2 (3.9%)	0 (0%)	0.50
Cup placement position error			
Horizontal position error (mm)	3.1 ± 1.8	2.3 ± 1.9	0.030
Vertical position error (mm)	2.5 ± 2.5	1.9 ± 1.2	0.12
Anteroposterior position error (mm)	2.7 ± 2.2	2.6 ± 1.7	0.80

CTN, computed tomography-based navigation; RI, radiographic inclination; RA, radiographic inclination

The horizontal position error was significantly larger in the portable CTN group at 3.1 ± 1.8 mm, compared to 2.3 ± 1.9 mm in the CTN group (*P* = 0.030). The vertical position error was 2.5 ± 2.5 mm in the portable CTN group and 1.9 ± 1.2 mm in the CTN group, while the anteroposterior position error was 2.7 ± 2.2 mm in the portable CTN group and 2.6 ± 1.7 mm in the CTN group. There were no significant differences in the vertical or anteroposterior position errors.

Regarding operative outcomes, the mean operative time was 99.3 ± 43.5 min in the portable CTN group and 104.6 ± 34.7 min in the CTN group. The intraoperative blood loss was 351 ± 202 g in the portable CTN group and 420 ± 204 g in the CTN group. No significant differences were observed in either of the parameters. The preoperative JOA scores were 57.3 ± 15.6 in the portable CTN group and 55.0 ± 14.4 in the CTN group, and the postoperative JOA scores at 1 year were 89.4 ± 9.0 and 89.5 ± 8.9, respectively, with no significant differences between the groups. In the CTN group, no significant difference was observed in the JOA scores between the 1-year follow-up (89.5 ± 8.9) and the final follow-up (90.2 ± 9.0). Regarding complications, the portable CTN group had one case of infection and one of dislocation, whereas the CTN group had one case of infection. However, the complication rates did not significantly differ between the groups (Table 3).

**Table 3:** Surgical and clinical outcome

	Portable CTN group	CTN group	*P*-value
Operative time (min)	99.3 ± 43.5	104.6 ± 34.7	0.50
Intraoperative blood loss (g)	351 ± 202	420 ± 204	0.090
Preoperative JOA score	57.3 ± 15.6	55.0 ± 14.4	0.47
Postoperative JOA score at 1 year	89.4 ± 9.0	89.5 ± 8.9	0.93
Complications			
Infection	1	1	> 0.99
Dislocation	1	0	0.50
Intraoperative fracture	0	0	> 0.99
Postoperative fracture	0	0	> 0.99
Nerve injury	0	0	> 0.99

CTN, computed tomography-based navigation; JOA, Japanese Orthopaedic Association

## Discussion

In this study, 51 hips that underwent THA using portable CT-based navigation were compared with 51 hips that underwent THA using conventional CT-based navigation. The portable system showed a significantly greater navigation error in anteversion and a higher number of cases with an error of 5° or more. A significant discrepancy in cup placement was observed in the horizontal direction. However, no significant differences were observed between systems in the operative time, blood loss, or postoperative clinical outcomes at 1 year.

The accuracy of CT-based navigation has been reported with errors typically ranging from 1.2° to 2.5° for RI and 1.0° to 3.4° for RA, at an average of approximately 2° [7, 14, 19–22]. Consistently, in the present study, the navigation errors for conventional CT-based navigation were 2.2° for RI and 2.3° for RA. However, portable CT-based navigation was inferior to the conventional system regarding RA, with a higher proportion of cases with a ≥ 5° navigation error. Potential reasons include the reduced field of view of the portable infrared camera, decreased camera precision, and slower processing speeds of the main unit. These challenges can be addressed through future hardware and software advancements in portable CT-based navigation systems. Despite these technical limitations, the clinical performance of portable CT-based navigation was comparable to those reported for other portable navigation systems [23, 24].

Regarding placement errors in CT-based navigation, discrepancies of 1.5–3.2 mm horizontally, 1.3–2.8 mm vertically, and 1.5–2.8 mm in the anteroposterior position, have been reported [15, 17, 19, 20, 22]. Comparably, in the present study, the corresponding errors were 2.3 mm, 1.9 mm, and 2.6 mm, respectively. Although the portable CT-based navigation system is inferior in terms of positional accuracy in the horizontal direction, it allows the surgeons to confirm the cup position during placement in real time, which is not possible with conventional systems. This offers a significant benefit as it enables inexperienced surgeons to maintain an appropriate acetabular offset and preserve adequate bone stock [21].

There were no significant differences between portable and conventional CT-based navigation regarding operative time or blood loss. THA using conventional CT-based navigation results in longer operative times than conventional THA procedures because it requires intraoperative registration for surface matching [25, 26]. As the portable CT-based navigation system requires the same procedure, no difference was observed between the two groups. Furthermore, no significant differences were observed in functional outcomes or complications 1 year postoperatively. Consistent with previous reports, this may be because the accuracy of implant placement was not directly reflected in improved clinical outcomes in the short term [27, 28]. In Japan, regarding cost-effectiveness, the portable system involves a per-case rental fee of roughly $350 and $200 for consumables, in contrast to the conventional system’s initial installation cost of about $220,000. Considering the comparable functional outcomes, the portable CT-based navigation system represents an economically efficient alternative with notable clinical utility.

This study had some limitations. First, the follow-up period was limited to one year. Although no significant differences in clinical outcomes were observed within this period, this duration is insufficient to draw definitive conclusions regarding long-term clinical equivalence. Second, although a power calculation was performed, the small sample size may have been insufficient to detect significant differences in the clinical outcomes or complication rates. Third, while the study was limited to Crowe type I hip arthritis, the retrospective design may have introduced bias regarding the severity of the preoperative deformity, potentially affecting placement accuracy. Fourth, the implant types were not standardized between the portable and conventional CT-based navigation groups as implant choice was limited by navigation system compatibility, which could have influenced the cup stability and clinical results. Fifth, although we attempted to minimize selection bias by allocating patients in a quasi-random manner among four available navigation systems, the lack of a prospective randomized design may still have introduced potential selection bias. Nevertheless, a major strength of this study is that it is the first to compare portable and conventional CT-based navigation systems at a single institution, potentially providing valuable insights for hip surgeons.

In conclusion, the portable CT-based navigation system is currently inferior to conventional CT-based navigation systems in terms of accuracy, placement angles, and positioning. However, no significant differences were found between systems regarding operative time, blood loss, or clinical outcomes 1 year postoperatively. Future research should target advancements in the hardware and software of portable systems to improve their accuracy and clinical utility.

## Data Availability

No datasets were generated or analysed during the current study.
